# A Masterly Hospital History

**Published:** 1917-12-01

**Authors:** 


### THE INSTITUTIONAL LIBRARY.

### A MASTERLY HOSPITAL HISTORY.

### The Smiths of Bristol Royal Infirmary.

DR. G. MUNRO SMITH, who died last January, deserves to be remembered among hospital historians, and the bulky volume,* which he lived to pass for the press but not to publish, is a fine memorial to his hospital and to his work. The Bristol Royal Infirmary, whose story is here unfolded, claims a precedence as one of the oldest of provincial hospitals, and has attracted a remarkable series of devoted and able men. By one of those fortunate chances, which come to those who deserve them and to no one else, Dr. Smith completed his book in 1914. No more convenient date for closing a chapter could be found, since a contemporary of the war could have hardly failed to regard his institution's work, as a branch of the 2nd Southern General Hospital, as dwarfing its previous history. The truth is all the other way. Its work as a war hospital is a chapter; its previous achievements laid the foundation which made that chapter possible. Nor is this fact the only one in which the late Dr. Smith may glory. He tells us that the Bristol Royal Infirmary already possessed fourteen bulky volumes of "Biographical Memoirs," which differ from a collection of press-cuttings in the same way that Pepys's Diary differs from the papers in the Record Office.

These "Biographical Memoirs" were compiled by Richard Smith, who was surgeon to the infirmary from 1796 to 1843. He was a man who had the wit to see that personal detail was the tissue of public history. He therefore gave not only a list of officers and minutes, but a sketch of each man's dress and mannerisms that far excels the array of board-room portraits on which alone many hospitals have to rely. He had a gift for that kind of irrelevance which is so much more important than unadulterated fact. His portraits are neither skeletons, stripped of flesh and blood, nor tailors' models. He saw that a hospital was the centre and focus of the life of the district which it serves, and that what is in men's thoughts is no less important than what men do. So his records are not obituary notices. They are as near the living tissue as he could contrive to make them, and the story of the infirmary in the eighteenth century is that, too, of the social and literary life of Bristol at this epoch. He knew, in fine, that the history of a hospital is the history of the men who make it.

With such material at his disposal Dr. Munro Smith could hardly write a dull book. Indeed, here and there his has been the odious task of the expurgator, for the truth, especially in small matters, is rarely considered fit for publication. For some of Smith's delightful portraits our readers must turn to the volume itself, and when they have done so they will hardly deny that, for instance, Dr. Logan's habitual costume of roquelaure, wig, and rapier is no less important to posterity than his services to medical science. At that date married apothecaries were rejected as unqualified persons, and the matron received a salary of £15 a year. At the first annual dinners the proceeds did not always cover the expenses, and the modern plan of securing subscriptions from those who do not intend to dine was unknown—indeed, unthought of. On the other hand, the affairs of the infirmary were discussed so heartily that on one occasion Dr. Rigge used his cane more freely than any other argument, and, in consequence, it was decided that the only business to be discussed at these dinners should be the food. In fact, by 1780 the dinners ceased. The sermons which preceded the dinners were also very much to the point, since, for some reason, the chaplaincy was a perpetual source of squabble among the subscribers, and the temptation to make a good hit from the pulpit was too strong to be missed. The Rev. Josiah Tucker also did not spare to scourge "the masses" on these occasions, and attributed their licentiousness, much in the vein of Coventry Patmore, to the extension of the franchise. The visitors in the early days took their duties gravely, and animadverted seriously on the quality of the beer supplied.

We pass reluctantly from these early pages to later matters, not because the fund of humour is exhausted, but because only by some sample of the treatment can we give any idea of the spirit of the book. There is, indeed, hardly a dull minute in the dreariest report of any hospital which is not the expression of a certain tendency of social life, and it has been the recognition of this fact which gives this volume its value and its distinction as a history. When duelling gave way to arbitration, and arbitration money was often deposited in some treasurer's hands, a new source of income was abated, and, in the same way, if the growth of workmen's subscriptions and representation is in future to be understood, how invaluable would be a contemporary record of board-room bickerings and of the personalities of the delegates and the board which first considered their election! Again, the days of apothecaries' apprentices seem remote, but how typical of the life of the medical profession to-day would be a picture of the years spent by young medical men in waiting for a place upon the staff! This is the real lesson to be learnt from this book, and it is noteworthy that the details on which we have lingered are not imported by any desire for local colour, but grow like moss upon the stones of the building whose story is being told. For

* *A History of the Bristol Royal Infirmary.* By G. Munro Smith, M.D. Bristol: T. W. Arrowsmith, Ltd. London: Simpkin, Marshall. Price 12s. 6d. net.

**THE INSTITUTIONAL LIBRARY**—(*continued*).

instance, the account of the quarrel between the committee and the physicians, which is given in Chapter X., is alike one which no sensible man would miss for its telling and that no hospital worker can neglect for its facts. The protagonists here, as elsewhere, have their paragraph of biography, and the more remarkable, like Dr. Rigge, we come to know as fully as those who live in the pages, not of history, but of the finest fiction. So early as 1790 the infirmary sought to increase its income by a performance of "extraordinary amusements," and people came in a crowd to see "the child of promise stand on her head upon the point of a spear," to the relief of the hospital's treasurer. We learn, too, of an ingenious plumber, who invented the principle of the water ram, which has since developed into the improved apparatus familiar to sanitary engineers. When the west wing was being built in 1806 many suggestions were made for the improvements which it should embody. These have a significance that needs no emphasis. They included bathrooms, with a hot-water supply; the numbering of the wards; a patient's dining-room, and a chapel.

On the early difficulties of *post-mortem* examinations and of teaching anatomy, on the complaints of out-patients, on the attempts of unattached practitioners to attend the consultations of the staff, on the past medical fashions—inhalations of cows' breath, for example—it would be pleasant to dwell: they form part of the tissue of the story.

In the delightful eighteenth chapter, Dr. Munro Smith explains his method, which, indeed, can stand in no need of defence to anyone who has once dipped into his book. The dinners and toasts of the first half of the nineteenth century, which usually preceded or concluded business, are vividly described, and we cannot forbear to mention the curious toast drunk in 1839 to Princess Charlotte. It ran: "To the Princess Charlotte, and may she know how to prize the sweets of liberty by an early confinement." The club life of Bristol, in which the infirmary's staff took an active part, makes amusing reading. Even when the author comes to the end of the period preserved so faithfully in Richard Smith's biographical memoirs, his perception does not fail him, though he has to be more sparing of detail than he need be in relation to the personalities of the past.

The later development of the Royal Infirmary, especially its expansion under the adventurous financial policy of Sir George White, has been described so fully in THE HOSPITAL that we need not record it here. Our aim has rather been to sketch the method which has made Dr. Munro Smith's history the most original and the most interesting of its class. For, to the best of our knowledge, no other hospital has produced a volume to compare with it, either in range, or wealth of detail, or value to posterity. It is the growth of the voluntary system that we study in these vivid pages, for the example perfectly seen becomes the type; and no such capable guide as Dr. Smith have we met elsewhere, nor one so worthy of his subject and of the opportunity created by the researches and the penetration of Richard Smith, the Boswell of hospital literature.

---

**An Ethical System: Based on the Laws of Nature.** By M. DESHUMBERT. Translated from the French by LIONEL GILES, M.A., D.Litt. (The Open Court Publishing Company. Price 2s. 6d.)

THIS book, simple in theme if elaborate in structure, is really an exposition of the bearing of the evolutionary theory upon that summed up so finely by the late Samuel Butler: "The true laws of God are the laws of our own well-being." To arrive at the latter M. Deshumbert induces from the study of living organisms certain aims which may be summarised successively as fertility and intelligence. The end is life itself. The means are fertility of offspring and intelligence rather than force. From the habits of plants and animals the writer then induces what he calls maternal morality: the desire of the organism rather for the well-being of its offspring than for its own survival. From the habits of gregarious animals the first example of tribal morality is inferred; from those of the inevitable bee the morality of the citizen is induced. At this point, in short, we arrive at the central postulate of Aristotle. The factors of co-operation and union are then illustrated, and it is then easy to state that the aim of Nature is "life reaching the highest possible pitch of activity, morality, and intelligence."

A more interesting section of the book deals with objections to this theory. Here the inadequacy of the author's defence becomes increasingly evident. It must suffice us to say that a man intelligent enough to see that death is as necessary to life as birth, should yet declare that it is unnatural (if not worse) to drink wine. He declares that Nature makes not wine, but grapes, which would imply that the man who makes the wine is not a part of Nature. The conclusions are so trite that the popularity of the book is easily explained. The writer has compiled a superstructure without thinking out its implications.

**Glaucoma: A Handbook for the General Practitioner.** By R. H. ELLIOT. (London: H. K. Lewis and Co., Ltd. Pp. 60. Price 3s. 6d. net.)

As a short and comprehensive introduction to the subject of glaucoma, for the use of students of the science of ophthalmology, we have nothing but praise for this handbook. Its author has succeeded in compressing the salient facts and the most widely accepted theories concerning an exceptionally complex and elusive problem into a comparatively small space. His arguments are clearly stated, and ought to be comprehensible to anyone who has some previous clinical knowledge of the eye and its diseases.

The value of a book of this kind to the busy general practitioner, who has neither time nor opportunity to become a specialist, is, in our opinion, a debatable question. Responsibility for the treatment of serious eye disease must always rest with the ophthalmic surgeon alone, and the general practitioner is chiefly concerned in the question of differential diagnosis, a subject to which but little space has been devoted in the handbook. This may be because the author is of opinion that accurate differential diagnosis of eye disease, more especially of glaucoma, can only be learnt by practical experience, in which case we fully agree with him.

